# The Regulation of Light Sensing and Light-Harvesting Impacts the Use of Cyanobacteria as Biotechnology Platforms

**DOI:** 10.3389/fbioe.2014.00022

**Published:** 2014-07-01

**Authors:** Beronda L. Montgomery

**Affiliations:** ^1^Plant Research Laboratory, Department of Energy, Michigan State University, East Lansing, MI, USA; ^2^Department of Biochemistry and Molecular Biology, Michigan State University, East Lansing, MI, USA

**Keywords:** biotechnology, cyanobacteria, light signaling, photoinhibition, photosynthesis, phycobilisomes, synthetic biology, systems biology

## Abstract

Light is harvested in cyanobacteria by chlorophyll-containing photosystems embedded in the thylakoid membranes and phycobilisomes (PBSs), photosystem-associated light-harvesting antennae. Light absorbed by the PBSs and photosystems can be converted to chemical energy through photosynthesis. Photosynthetically fixed carbon pools, which are constrained by photosynthetic light capture versus the dissipation of excess light absorbed, determine the available organismal energy budget. The molecular bases of the environmental regulation of photosynthesis, photoprotection, and photomorphogenesis are still being elucidated in cyanobacteria. Thus, the potential impacts of these phenomena on the efficacy of developing cyanobacteria as robust biotechnological platforms require additional attention. Current advances and persisting needs for developing cyanobacterial production platforms that are related to light sensing and harvesting include the development of tools to balance the utilization of absorbed photons for conversion to chemical energy and biomass versus light dissipation in photoprotective mechanisms. Such tools can be used to direct energy to more effectively support the production of desired bioproducts from sunlight.

## Introduction

The use of photosynthetic organisms as biotechnological platforms for the production of bioproducts has gained significant interest in recent years. These organisms have the potential to combine the functions of photocatalyst and production platform in a single organism, thereby potentially truncating the process from harvesting solar energy to the production and isolation of important bioproducts, including biofuels (Lindberg et al., 2010; Melis, 2012; Oliver and Atsumi, 2014). The general potential of cyanobacteria as biofactories or for biotechnological applications has been addressed in recent reviews (Lindblad et al., 2012; Oliver and Atsumi, 2014).

Cyanobacteria and algae have been described as better photosynthetic platforms than land plants due to high photosynthetic rates and a greater potential for the diversion of photosynthetically fixed carbon to the production of target molecules (Melis, 2009). This higher potential is primarily due to there being no need to divert energy to support non-photosynthetic tissues, which can comprise a significant proportion of the organism in the case of plants (Melis, 2012). Although still somewhat early in their development as biotechnology chassis, some developmental or engineering modifications have been tested for improving the use of cyanobacteria as effective production platforms. Increasing the partitioning of photosynthetically produced carbon to pathways of interest can increase overall yield of the production of target molecules in engineered cyanobacteria. Cyanobacteria can be engineered to couple the expression of genes that increase flux through pathways which provide key substrates or precursors to engineered pathways to further promote the production of target molecules (Bentley et al., 2014; Halfmann et al., 2014; Kiyota et al., 2014). Alternatively, knocking out natural carbon sinks or storage products, such as glycogen synthesis, can result in a greater proportion of carbon partitioning to non-native products (Li et al., 2014; van der Woude et al., 2014).

Oxygenic photosynthesis has been proposed to be theoretically limited to an 8–10% efficiency of solar-to-biomass energy conversion (Melis, 2009). This theoretical limit can be attained under low light conditions, but drops to 2–3% or even lower for some species in full sunlight (Melis, 2012). Sub-optimal light, light-harvesting complexes that are not in balance with the external environment, or photoinhibitory mechanisms associated with excess light absorption are among the factors that can contribution to this limitation. One mechanism for dissipating excess light is non-photochemical quenching (NPQ), which is the dissipation of excess energy as heat (Niyogi and Truong, 2013). NPQ is the primary mechanism that appears to limit solar-to-biomass conversion efficiency (Melis, 2012). This observation suggests NPQ regulation as a potential target for improving the development of cyanobacteria as bioproduction strains. Other targets for improving biotechnological or industrial applications using these organisms have included assessing the impact of source–sink relations on productivity. Prior studies indicate that engineered cyanobacterial strains which have an additional sink for carbon, either through incorporation in particular products or through export of carbon-based compounds from the cell, respond by exhibiting increased rates of carbon dioxide assimilation and increased rates of photosynthesis (Ducat et al., 2012; Ungerer et al., 2012; Oliver et al., 2013; Bentley et al., 2014; Halfmann et al., 2014).

The ability to photosynthesize and adapt to variable environments are the primary reasons that cyanobacteria exhibit great potential for bioengineering and biotechnological applications. However, some potential damaging impacts of light or light-dependent utilization of energy for acclimation responses must be balanced to maximize the efficacy of cyanobacteria as production chassis. The content and number of phycobilisomes (PBSs), i.e., accessory photosynthetic light-harvesting complexes, and core photosystems have to be balanced to maximize light absorption for the conversion of light energy to chemical energy in the form of photosynthate, while also minimizing the absorption of excess light. Excess photoexcitation can lead to photoinhibition and phototoxicity and thereby limit the production of target molecules. The utilization of light in photomorphogenic or light-dependent growth and developmental responses, for which the fitness implications are still not well defined, can be quite costly energetically and thus could impact organismal productivity. Thus, the impact of light on additional phenotypes requires attention as these light-dependent processes can contribute to or draw away energy available for the improvement of solar-to-biomass conversion efficiencies or conversion of photons to chemical energy used to support production of desired compounds.

## Light Impacts on Culture Growth and Productivity

**Figure 1:**
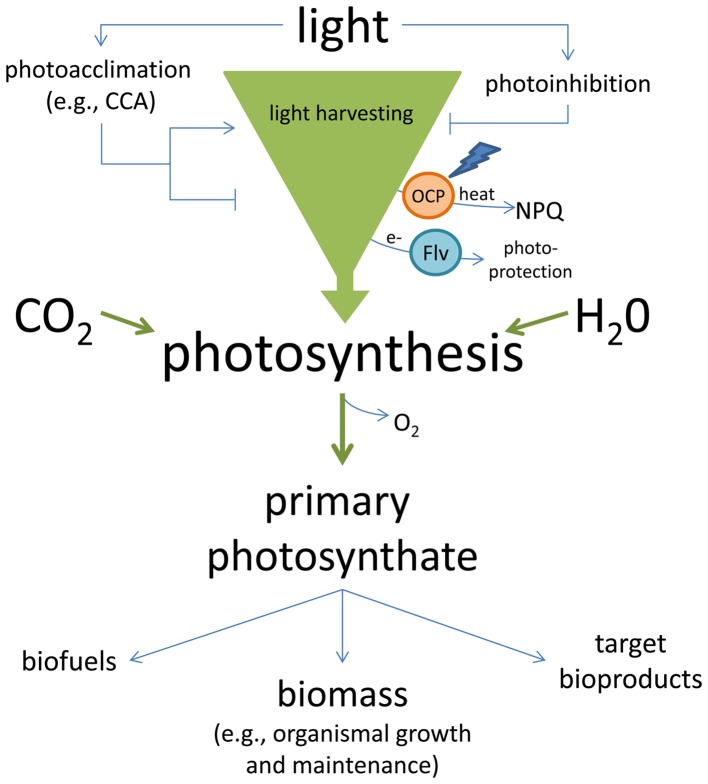
**Light-harvesting in cyanobacteria and potential costs and/or contributions to solar energy conversion**. Light harvested in cyanobacteria can be used with carbon dioxide (CO_2_) and water (H_2_O) for photosynthesis. When excess energy is absorbed, it can result in damage to the photosystems and photoinhibition, or energy can be dissipated as heat by proteins such the orange carotenoid protein (OCP) or electrons can be transferred to alternative acceptors such as flavodiiron proteins (Flv) during photoprotection. In response to the environment, acclimation responses which tune the size and pigment composition of photosystems to maximize light absorption, or alter the size and composition of light-harvesting complexes to reduce or increase light absorption, can modify the organismal potential for light-harvesting. Light energy that is used for the reactions of photosynthesis can be converted to chemical energy (primary photosynthate) that supports growth and maintenance, including biomass production, or can be diverted and used for the production of target bioproducts and/or biofuels.

The importance of light both in providing energy to fuel the growth of cyanobacterial and algal strains and the bioproduction of target molecules, as well as the potential damaging effects of excess light has been addressed, primarily from the perspective of minimizing shading and maximizing productive exposure of all cells in a culture to light (Scott et al., 2010). However, the impact of light on cultures can be quite complex in that light can initiate acclimation responses in addition to primary photochemistry. Acclimatory light responses can either increase or decrease overall cellular productivity (Figure 1).

### Light-regulated transcriptional and cellular responses impact energy production capacity in cyanobacteria

#### Light impacts cyanobacterial photosynthetic complex composition and cellular physiology

The acclimation response, historically known as complementary chromatic adaptation (CCA), is one process by which cyanobacterial cells use a transcriptional response to produce PBSs that are spectrally tuned to maximally absorb the prevalent wavelengths of light in the ambient environment (Gutu and Kehoe, 2012). CCA also drives light-dependent alterations of cellular or filament morphology (Bennett and Bogorad, 1973; Bordowitz and Montgomery, 2008). CCA, thus, through tuning light absorption to the external environment maximizes the potential for light energy to chemical energy conversion. Notably, CCA itself is under the regulation of a light-sensitive photosensory protein, i.e., RcaE, which controls the transcription of PBS-encoding genes (Kehoe and Grossman, 1996; Terauchi et al., 2004) and light-dependent morphological changes (Bordowitz and Montgomery, 2008; Singh and Montgomery, 2014). A mismatch between PBS protein composition with light available results in an initial loss of photosynthetic efficiency until the protein composition is recalibrated with the predominant wavelengths of light available (Campbell, 1996).

A significant contribution of energy to altering other aspects of growth and development occurs in response to changes in the external light, including morphological phenotypes. Changes in the prevalent wavelengths and intensity of light can lead to CCA-associated shifts between spherical and rod-shaped cells (Bennett and Bogorad, 1973; Bordowitz and Montgomery, 2008; Pattanaik et al., 2012; Walters et al., 2013). Distinct wavelengths have also been shown to induce cellular differentiation in some cyanobacteria from vegetative cells to other cell types including motile hormogonia (Damerval et al., 1991) or spore-like akinetes (Thompson et al., 2009). These morphological changes have largely unknown impacts on efficiency and solar energy conversion. Whereas, the ability of cells to modulate photosynthetic pigment composition is beneficial to long term culture productivity, the energetic costs or benefits of photo-dependent morphological control is less well understood. It has been suggested, however, that the morphological effects may be related to controlling cell volume and the related capacity for thylakoid membrane content, which drives PBS quantity primarily for adaptation to low light conditions (Montgomery, 2008; Pattanaik et al., 2012; Walters et al., 2013). Thus, the morphological alterations may regulate photosynthetic membrane capacity and thereby contribute to optimizing photosynthetic efficiency in response to changes in the external environment.

Similar to the benefits of regulating PBS protein or pigment composition, distinct photosystem (PS) proteins may also be used under different environmental conditions. Such alterations occur as a part of acclimation responses that are generally associated with fitness advantages under particular conditions, which include differences in light quality and/or intensity (Vinyard et al., 2013, 2014). In addition to protein substitutions or modifications, the overall antenna size may be modulated to alter cellular productivity and/or for cellular photoprotection. There are noted correlations of photosynthetic light-harvesting complex size and number with photoacclimation or photoprotection responses (Jodłowska and Latała, 2013). Diel (Jacquet et al., 2001; Poulin et al., 2014) and circadian cycles (Cervený and Nedbal, 2009) can also impact chlorophyll levels, which can be directly related to potential productivity and/or photoinhibition. Such acclimation responses suggest strong effects of growth conditions on the potential productivity of strains. These acclimation responses can either increase light-harvesting for photosynthesis or alternatively can promote photoprotection, which limits damage while often simultaneously reducing photons harvested for the photochemical reactions of photosynthesis (Figure 1).

The production of different versions of light-absorbing photosynthetic proteins or modulations of antenna size, in addition to other light-dependent changes in growth and development or photomorphogenesis, require energy that is spent during the acclimation responses – energy that is no longer available for the production of target molecules or products. To address this potential non-productive use of energy during acclimation, some analyses have been conducted to assess the benefits of truncating antennae size on supporting an overall increase in photosynthetic productivity for dense mass culturing. Truncated antennae promote light penetration and absorption for photosynthesis, while simultaneously reducing the potential for photoinhibition (reviewed by Melis, 2009).

#### Light impacts cyanobacterial transcription through the regulation of sigma factors

Sigma (sig) factors that impact cyanobacterial gene transcription are themselves regulated by environmental factors (Imamura and Asayama, 2009). For example, Sig B levels increase in response to stress, including under heat shock, in response to nitrogen starvation and in darkness (for review see Imamura and Asayama, 2009). Analysis of a Δ*sigB* mutant in *Synechocystis* sp. PCC 6803 (hereafter *Synechocystis*) indicated that SigB serves as a negative regulator of the expression of a number of photosynthesis-related genes, among others (Foster et al., 2007). Thus, SigB accumulation under stress conditions likely contributes to downregulation of photosynthetic capacity, and thus could have a negative impact on photosynthetic capacity when overexpressed. Some cyanobacterial sigma factors also have been reported to be involved in the circadian regulation of rhythmic gene expression (Tsinoremas et al., 1996; Nair et al., 2002). The accumulation of others is regulated by light, including SigB, SigD, and SigE (Imamura et al., 2003a; Tuominen et al., 2003). These light-induced sigma factors have been shown to impact the transcription of light-induced PBS and PS genes (Imamura et al., 2003a; Summerfield and Sherman, 2007; Yoshimura et al., 2007; Pollari et al., 2009). SigB and SigD have also been shown to have a role in cellular adaptation of *Synechocystis* to high light conditions (Imamura et al., 2003b; Pollari et al., 2008, 2009, 2011), as has a SigD homolog in *Synechococcus elongatus* PCC 7942 (Seki et al., 2007). Recent studies with *Synechocystis* strains in which several *sig* genes were deleted highlighted an additional role of Sig proteins in modulating light use efficiency in response to moderate changes in light intensity (Tyystjärvi et al., 2013). Several sigma factors are, thus, directly linked to regulation of photosynthetic potential and the capacity for phototoxicity.

The correlation of several sigma factors with the regulation of photosynthesis-related genes and light use efficiency suggests that modulation of these genes could impact the development of cyanobacterial strains as production platforms. In this regard, *sigB* overexpression has been shown to improve the use of *Synechocystis* as a platform for producing the biofuel butanol, as well as for improving tolerance to non-optimal temperature and reducing reactive oxygen species (ROS) generation (Kaczmarzyk et al., 2014). By comparison, *sigE* overexpression negatively impacts the expression of PS genes and results in reduced photosynthetic efficiency in nutrient-replete growth (Osanai et al., 2013). Thus, the regulation of sigma factors *in vivo* has complex outcomes. Apart from photosynthesis-related genes, there may well be other non-photosynthetic genes whose expression is modulated by environmental regulation of sigma factors that could be vitally important for maximizing solar energy conversion and the development of cyanobacteria as biotechnological platforms. The utilization of systems biological approaches, including transcriptomics and proteomic analyses, to explore such connections could prove vitally important for further development of cyanobacterial production systems that have defined means for tuning gene expression over a wide range of conditions to promote robust and sustainable production of value target molecules.

### Photons that are not converted to chemical energy limit production and can cause cellular damage

Unless regulated, excess or variable light can lead to photoinhibition and the potential generation of damaging compounds. Phototoxicity is induced by high light, fluctuating light, and nutrient deficiencies that can impact the abundance and composition of light-harvesting complexes. An imbalance between light-harvesting complexes and the quality and/or quantity of available light can result in the generation of ROS, which can lead to nucleic acid and protein damage and lipid peroxidation (reviewed by Blokhina et al., 2003). There are several known physiological mechanisms for dealing with potential phototoxicity, including photoinhibition and regulating the amount of light transferred to the photosystems through dissipating excess absorbed light energy. Photoinhibition results from a reduction in photosynthetic efficiency due to light-induced damage of photosystems (PSs), primarily PSII (Tyystjärvi, 2008). Excess light is predominantly dissipated through defined mechanisms such as NPQ or loss of excess energy as heat or fluorescence (Niyogi and Truong, 2013) (Figure 1). Dissipatory regulation of light primarily occurs through state transitions and protein-mediated photoprotective mechanisms.

#### Dissipation of excess light through state transitions

State transitions regulate the amount of energy transferred from PBSs to the photosystems through physical movement and/or disruption of the association of the PBS with PSI or PSII at the thylakoid membrane surface (Joshua and Mullineaux, 2004). State transitions, which are defined as State 1 or 2, allow a rapid mechanism for regulating light absorption by the photosystems. In State 1, energy from PBSs is preferentially transferred to PSII; however, when light absorption exceeds the capacity of PSII excitation, a transition to State 2 occurs during which energy from PBSs is funneled to PSI (Mullineaux and Emlyn-Jones, 2005). Traditionally, lateral mobility or diffusion on the thylakoid membrane surface of PBSs between PSI and PSII reaction centers has been described as a mechanism for state transitions in cyanobacteria. Recently, a cyanobacterial megacomplex, which contains a PBS complex together with both PSI and PSII has been described (Liu et al., 2013). The megacomplex is still proposed to function to transfer energy to either PSI or PSII likely through distinct mechanisms (Liu et al., 2013). If the occurrence of this megacomplex is widespread in cyanobacteria, a revision of exactly how state transitions occur may be necessary as the need for lateral diffusion of PBS for state transitions may not be required. The coordination or control of PBS energy transfer to PSII vs. PSI is one significant point of regulation of excitation energy transfer and its conversion to chemical energy. Thus, the regulation of state transitions has the potential to drive the balance of photosynthetic energy transfer or energy dissipation for fine-tuning the conversion of chemical energy to biomass.

#### Photoprotective mechanisms used to protect against excess light absorption

One mechanism for the dissipation of excess absorbed light energy depends on the function of the orange carotenoid protein (OCP) and its accessory protein fluorescence recovery protein (FRP). OCP and FRP work together to regulate energy flow from the PBS to PS in a NPQ of energy (Kirilovsky and Kerfeld, 2012, 2013). OCP is a carotenoid-bound photoactive protein that is activated by blue light (Figure 1). Photoactivated OCP binds to the PBS core and converts absorbed light energy to heat, thereby diverting energy transfer to the photosystems (Kirilovsky and Kerfeld, 2012, 2013). FRP releases activated OCP from the PBS and converts it back to its ground state, thereby resetting the system (Kirilovsky and Kerfeld, 2013). OCP has also been recently shown to have an independent role in protecting cells from strong light-induced single oxygen production, which is distinct from its role in binding to PBSs (Sedoud et al., 2014).

Another cyanobacterial photoprotective mechanism to divert potential overexcitation of the PSs involves the flavodiiron (Flv) proteins. These proteins function in removing electrons from the electron transport chain and transferring them to alternative electron acceptors (for review see Mullineaux, 2014) (Figure 1). The expression of *flv* genes is regulated by environmental factors including increased light intensity (Zhang et al., 2009; Bersanini et al., 2014) and fluctuating light (Allahverdiyeva et al., 2013). A greater understanding of the regulation of OCP and Flv systems may allow targeted induction of these systems to support the efficacy of cyanobacteria as biotechnological chassis.

## Implications of Light-Associated Growth Responses on the Use of Cyanobacteria as Production Strains

### Current limitations associated with light responses in cyanobacterial production strains

Increasing carbon sink strength and/or adding additional carbon sinks to alter source–sink relationships and potentially relieve sink-related feedback inhibition of photosynthesis have been attempted to improve productivity in cyanobacterial strains. Current tools for increasing the production of soluble sugars in cyanobacteria include subjecting cells to stresses such as high salt concentrations in growth media (Ducat et al., 2012; Du et al., 2013; Hays and Ducat, 2014). However, such conditions often lead to reduced accumulation of photosynthetic pigments and associated reductions in growth (e.g., Kanesaki et al., 2002; Marin et al., 2004; Bhadauriya et al., 2007; Singh and Montgomery, 2013). For cells subjected to stress, exposure to excess light can lead to the production and accumulation of damaging ROS. Therefore, subjecting cells to stress as a means to induce the accumulation of soluble carbohydrates has significant drawbacks. Nitrogen-limitation of *Synechococcus* sp. PCC 7002 has been used to increase the carbon-to-nitrogen ratio and glycogen content to improve its utility as a biomass feedstock (Möllers et al., 2014). Nitrogen starvation of cyanobacteria leads to degradation of PBS complexes (Paone and Stevens, 1981; Stevens et al., 1981; Salomon et al., 2013). Under these conditions, cyanobacteria exhibit a reduction in growth and increased potential for the induction of photodamage similar to salt stress. Ultimately, the molecular bases of stress-related carbon allocation responses such as salt-induced soluble sugar production are often not well understood (Melis, 2013). Thus, knowledge-based attempts to genetically manipulate cyanobacterial strains to support increased production of target products are not possible to a significant degree.

### Beneficial light-associated properties of production strains

Production strains ideally will limit energy contributed to acclimation responses, particularly due to transient changes in the external environment, to maximize photosynthetic efficiencies – unless such an acclimation response is absolutely critical for survival. One method for avoiding the absorption of excess light that must be dissipated by NPQ and/or reducing the shading of cells in dense cultures or in benthic environments has been to isolate strains with smaller light-harvesting systems or truncated light-harvesting antenna (TLA) mutants (Kirst and Melis, 2014). These strains have been proposed to allow deeper penetration of light into cultures that should be associated with increased culture productivity (Melis et al., 1999; Melis, 2009; Kirst and Melis, 2014; Lea-Smith et al., 2014). Several methods have been used to identify or generate TLA mutants (Kirst and Melis, 2014); however, there has not been a straightforward association of such mutants with improvements in productivity (Blankenship and Chen, 2013). Recent studies indicate that interactions between light intensity and carbon availability may impact the degree of productivity for TLA mutants compared to wild-type cultures (Lea-Smith et al., 2014).

One suggested alternative to the creation of TLA mutants is to use cells that can absorb light in an expanded region of the visible spectrum to increase the total light that is used to drive photosynthesis and thereby overcome “shading effects” (Blankenship and Chen, 2013). One such possibility is to grow cells with distinct chlorophyll pigments that absorb distinct wavelengths of light in mixed culture to increase photon use efficiency (Scott et al., 2010). Beside the common chlorophyll *a* that is found in cyanobacteria, there are less common red-shifted chlorophylls. At least two red-shifted chlorophyll molecules, i.e., chlorophyll *d* and *f*, have been described (Swingley et al., 2008; Chen et al., 2010). Once the biosynthetic pathways of these red-shifted molecules are better understood, their synthesis may be introduced into a chlorophyll *a* containing strain using synthetic biology approaches to expand the range of visible light absorption supporting photosynthesis in a single organism. Another potential alternative is to grow organisms with distinct pigment compositions of the auxiliary PBSs or those that can adapt their PBSs to the external light conditions in mixed cultures. Alternate approaches require additional knowledge about the mechanisms used by cells to regulate antenna composition, size, and modulation *in vivo*.

## Conclusion

The increasing number of sequenced cyanobacterial strains should facilitate an increased understanding of regulatory and physiological mechanisms used by these organisms to adapt to variable environments. This knowledge may serve as bases for improved engineering and biotechnological adaptation in cyanobacteria (Jin et al., 2014). Although light sensing and light-harvesting have been the focus of the discussion here, carbon dioxide capture, fixation, and recycling are also targets or points of interest for maximizing the biotechnology applications for cyanobacteria (e.g., Ducat and Silver, 2012; Rosgaard et al., 2012). These areas and others will be equally benefited, and perhaps synergistically deployed, based on continued insights into the basic mechanisms and impacts of environmental variation on a range of cyanobacteria and the development of appropriate tools to improve adaptation of these strains as broadly applicable production platforms.

## Conflict of Interest Statement

The author declares that the research was conducted in the absence of any commercial or financial relationships that could be construed as a potential conflict of interest.
